# The effect of intravenous calcium administration on haemodynamics in perioperative cardiothoracic surgery and intensive care: A narrative review

**DOI:** 10.1016/j.ccrj.2026.100178

**Published:** 2026-04-18

**Authors:** Adam Miller, Jessica Omassoli, Joanna Simpson, Kyle Brooks, Aubrey Almeida, Ryan Ruiyang Ling, Ashwin Subramaniam

**Affiliations:** a Department of Intensive Care, Epworth Healthcare, Richmond, VIC, Australia; b Department of Anaesthesia, Peter MacCallum Cancer Centre, Melbourne, VIC, Australia; c University of Melbourne, Parkville, VIC, Australia; d Faculty of Medicine and Nursing, Monash University, Clayton, VIC, Australia; e Cardiac Sciences Clinical Institute, Epworth Healthcare, Richmond, VIC, Australia; f Yong Loo Lin School of Medicine, National University of Singapore, National University Health System, Singapore, Singapore; g Australia and New Zealand Intensive Care Research Centre, School of Public Health and Preventive Medicine, Monash University, Melbourne, VIC, Australia; h Department of Intensive Care, Dandenong Hospital, Monash Health, Dandenong, Victoria, Australia; i Peninsula Clinical School, Monash University, Frankston, VIC, Australia

**Keywords:** Cardiothoracic surgery, Calcium, Cardiothoracic intensive care, Vasopressors, Inotropes

## Abstract

Calcium plays an essential role in myocardial contractility, excitation-contraction coupling, and vascular tone. In cardiothoracic surgery, calcium supplementation is frequently administered during weaning from cardiopulmonary bypass (CPB) and in the early postoperative period to support haemodynamics. Despite its widespread use, the evidence base underpinning this practice remains limited and inconsistent. This narrative review explores the physiological rationale for calcium supplementation in the cardiothoracic surgical population, synthesises existing experimental and clinical data, and considers potential benefits and risks relevant to contemporary intensive care practice.

Animal models suggest that calcium desensitisation contributes to myocardial dysfunction following hypothermic circulatory arrest, with supplementation theoretically improving contractility. In human studies, calcium administration during CPB weaning or in the immediate post-CPB period has been associated with transient increases in mean arterial pressure, systemic vascular resistance and left ventricular stroke work index. However, these effects are short-lived and data beyond the early postoperative phase remains limited.

Potential risks of calcium supplementation include exacerbation of ischaemia-reperfusion injury, arrhythmogenesis, graft vasospasm, and tissue injury related to extravasation. The absence of specific guideline recommendations, in contrast to established consensus for vasopressor therapy, likely contributes to international variability in practice. Most studies are small, dated, or proof-of-concept and no high-quality randomised controlled trials have examined patient-centred outcomes such as vasopressor duration, organ dysfunction, or length of stay. Further multicentre observational and target-trial emulation studies are warranted to clarify the role of ionised calcium use in contemporary perioperative and intensive care practice.

## 1 Background

Each year, approximately 18,000 adults undergo cardiothoracic surgery in Australia and New Zealand, with more than 170,000 cases recorded in the Australia and New Zealand Society of Cardiac and Thoracic Surgeons (ANZSCTS) database.^1^ Postoperatively, approximately 70% of patients receive some level of inotropic or vasopressor support due to myocardial stunning, postoperative vasoplegia, or hypovolaemia.^2^^,^^3^ Vasoplegic syndrome, characterised by reduced systemic vascular resistance (SVR) and high-output circulation after cardiopulmonary bypass (CPB), occurs in up to 50% of patients, often requiring vasopressor support.[4], [5], [6]

The role of electrolytes in cardiothoracic surgery has been extensively investigated, with potassium and magnesium supplementation for the prevention and treatment of atrial fibrillation now established as routine practice by most members of the European Association of Cardiothoracic Anaesthetists^7^ and there are internationally published recommendations regarding their supplementation.^8^ In contrast, the role of calcium supplementation in influencing haemodynamic parameters remains unclear. Calcium chloride (CaCl_2_) contains approximately three times more elemental calcium than calcium gluconate, and no studies to date have directly investigated whether this difference translates into measurable haemodynamic effects.^9^ Animal studies have linked myocardial stunning to abnormal metabolism of calcium in the sarcoplasmic reticulum,^10^ yet evidence supporting calcium supplementation in cardiac-surgical patients is limited. Relatively few trials have investigated routine supplementation in this patient cohort, with conflicting recommendations regarding its benefit, and none have been conducted in an adult intensive care unit (ICU) setting or examined the effect on the total duration of vasopressor use.^11^^,^^12^

While guidelines provide recommendations for vasopressors after cardiothoracic surgery, none currently address the use of routine calcium supplementation.^13^ As a result, there is widespread variation between units and clinicians regarding calcium supplementation, with no consensus on ionised calcium (iCa) targets. An international survey across cardiothoracic centres found that calcium salts are routinely administered: 71% during CPB weaning, 78% to correct hypocalcaemia, and 54% after blood transfusions.^14^ Given these variations, we sought to investigate the relationship between calcium levels and the need for inotropes or vasopressor support, and to assess whether routine calcium supplementation may confer any clinical benefit in this patient population.

## 2 Methodology

To inform this narrative review, we performed a literature search across PubMed, MEDLINE, and Embase using combinations of keywords and MeSH terms relating to *calcium supplementation*, *cardiac surgery*, *inotropes*, and *vasopressors*. Search terms included: “calcium administration,” “ionised calcium,” “calcium chloride,” “calcium gluconate,” “cardiothoracic,” “cardiothoracic surgery,” “cardiopulmonary bypass,” “vasoplegia,” “vasopressor requirements,” and “inotrope support.” Additional terms such as “haemodynamics,” “perioperative,” “postoperative,” “contractility,” and “mortality” were also applied. Boolean operators were used to combine terms (e.g. *calcium AND cardiac surgery AND inotrope*; *ionised calcium AND vasopressor AND ICU*). The search was restricted to English-language publications without date limitations to capture both historical and contemporary evidence. Reference lists of relevant articles and recent guideline statements were also manually screened to identify additional studies.

Randomised controlled trials (RCTs), observational studies, cohort studies, case series, expert opinions, in vitro studies, and animal models exploring mechanistic effects were included. The initial database search yielded 223 records (PubMed = 170, MEDLINE/Embase (Ovid) = 53). After removing 46 duplicates, the remaining titles and abstracts were screened by an assessor for relevance to our topic. A total of 139 articles were excluded as irrelevant, and the remaining 36 full-text articles were assessed for eligibility.

Studies were included if they met the following criteria:

• Intervention/exposure: Administration of intravenous calcium supplementation or measurement of calcium in the perioperative or postoperative setting.
• Outcomes: Haemodynamic parameters, ionised calcium levels, ICU mortality, ICU length of stay, and inotrope or vasopressor usage/duration.

**Fig. 1:**
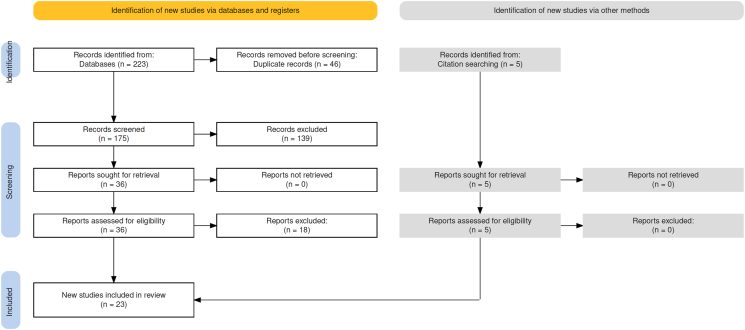
Literature search flowchart^15^.

Ultimately, 18 studies were included in the analysis. Reference list screening and citation tracking identified an additional five relevant articles, yielding a final total of 23 studies that were included in this narrative review (Fig. 1). Of these, five were conducted using in vitro or animal models, two were expert opinion pieces, and sixteen were human studies, of which four were paediatric populations. With the exception of the 2026 systematic review and paediatric studies, most of the available research originates from studies published prior to 2000. A summary of the literature is presented in Table 1.

**Table 1:** Literature review summary table.

Author(s)/year	Study type/methodology	Main topic	Relevance to calcium/vasopressors and inotropy	Subject type/size	Intervention(s)	Outcomes assessed	Relevant key findings
DiNardo, JA (1997)^11^	Expert opinion	Weaning from CPB	High/low	N/A	N/A	- N/A	- Argues for routine calcium use due to benefit in contractility and haemodynamics in ways not assessed by CI
Prielipp & Butterworth (1997)^12^	Expert opinion	Weaning from CPB	High/low	N/A	N/A	- N/A	- Challenges routine calcium use due to mild hypocalcaemia not impairing cardiac function and interactions with catecholamines and the potential to contribute to myocardial dysfunction
Banitt et al. (1993)^16^	In vitro	Relaxation responses of microvascular circulation of the heart	Moderate/low	Porcine hearts	Various vasodilatory agents	- Vascular reactivity to pharmaceutical agents postcardioplegia	- CCBs the most potent vasodilator of coronary arterioles postinduced cardioplegia in vitro samples - Diminished effects post cardioplegia
Choi et al. (2010)^17^	In vitro	Inotropy and relaxation	Moderate/low	Isolated rabbit hearts	ORG 30029, levosimendan, butanedione-monoxime and dopamine	- Isovolumetric left ventricular function - Myocardial oxygen consumption (MvO_2_)	- Calcium sensitisation improves systolic function and energy efficiency in postischaemic arrest in vitro samples but may exacerbate myocardial stunning - Ischaemia/reperfusion induced moderate cytosolic calcium overload
Grubb et al. (1999)^18^	In vivo animal	Haemodynamic effects of ionised calcium	High/moderate	6 anaesthetised horses	Intravenous calcium gluconate	- MAP, heart rate, mean PAP - CO/CI/SV - Right atrial pressure	- Calcium gluconate attenuated the anaesthetic-induced depression in cardiac index, stroke index, and maximal rate of increase in right ventricular pressure
Kumar et al. (2009)^10^	In vivo animal	Myocardial stunning and sarcoplasmic reticulum calcium uptake	High/low	In vivo pig models	Ischaemia-reperfusion plus Ca^2+^ tracing, glutathione depletion	- Ca^2+^ uptake - Contractile function	- Impaired sarcoplasmic reticulum calcium uptake correlates with contractile dysfunction
Rungatscher et al. (2013)^19^	In vivo animal	Postop myocardial dysfunction from DHCA	Moderate/low	In vivo Wistar rats	Levosimendan vs epinephrine vs placebo	- Myocardial function recovery post-DHCA	- Ca^2+^ desensitisation may support contractility post-DHCA in vitro samples
Murdoch et al.(1994)^20^	Interventional	CaCl_2_ postcardiac surgery	High/high	12 paediatric cardiothoracic patients	0.1 ml/kg CaCl_2_ 10%	- iCa, MAP, CI, SVRi	- CaCl_2_ increases MAP and SVRi with a decrease in CI
Averin et al. (2016)^21^	Retrospective observational	CaCl_2_ in paediatric cardiac ICU	High/high	68 paediatric cardiac ICU patients	CaCl_2_ infusions	- CO, HR, MAP - A-V oxygen saturation difference, serum lactate, cerebral rSO2	- CaCl_2_ increases CO, MAP, cerebral rSO2 and urine output - CaCl_2_ decreases A-V saturation difference, serum lactate, and heart rate
Karki et al. (2019)^22^	Retrospective observational	CaCl_2_ in paediatric acute heart failure	High/moderate	77 paediatric ICU patients	CaCl_2_ infusions with vasopressin	- sBP, dBP, OU, HR - Serum lactate, arterial pH base excess	- CaCl_2_ used concurrently with vasopressin may improve surrogates for CO and prevent organ dysfunction more than vasopressin alone
Kimura et al. (2018)^23^	Retrospective observational	Association of iCa and ICU LOS	High/low	357 paediatric cardiothoracic patients	N/A	- iCa (average, max and min) - ICU LOS	- Higher iCa within 24 h after congenital cardiac surgery using CPB was independently associated with longer LOS in the ICU
Auffant et al. (1981)^24^	Observational	Ionised calcium and haemodynamics	High/moderate	45 patients	N/A	- MAP, CI, SV, HR - Systemic and pulmonary vascular resistance - Ionised calcium	- Ca^2+^ supplementation did not significantly improve CI, SV, or SVRi beyond the first 2 h postcardiothoracic surgery. - Postoperative hypocalcaemia occurs following CPB but not enough to cause cardiovascular depression
Lomivorotov et al. (2020)^14^	Survey	Calcium use post CPB	High/high	Survey (100 centres)	Survey on Ca2+ bolus/infusion use	- Practice patterns	- Significant global variability in calcium use during CPB weaning - Noradrenaline is the most used inotrope/vasopressor
Egi et al. (2011)^25^	Retrospective observational	ICU calcium levels and mortality	High/low	7024 patients	N/A	- ICU mortality, - Ionised calcium levels	- Ionised calcium values < 0.8 mmol/L or >1.4 mmol/L were independently associated with ICU and hospital mortality.
Myles et al. (1993)^26^	Case-control	Low vascular resistance syndrome following cardiac surgery and CPB	Moderate/low	42 patients (including 84 controls)	Preoperative ACE-I or calcium antagonists	- Incidence of low SVR syndrome	- There was no association between therapy with calcium antagonists and the low systemic vascular resistance syndrome following CPB
Shapira et al. (1984)^27^	Prospective interventional	Haemodynamic effects of intravenous injection of calcium chloride	High/moderate	26 patients	CaCl_2_ bolus vs continuous infusion	- Myocardial contractile element velocity - Cardiac index, - Aortic blood flow - ECGs - Left ventricular, systemic arterial, pulmonary arterial, and left atrial pressures.	- Significant early haemodynamic improvement following CaCl_2_ - Improvement in cardiac index was not sustained for >5 min - CaCl_2_ bolus more effective than infusion
Kristof & Magder (1999)^6^	Prospective cohort	Low systemic vascular resistance (SVR) state post-CPB	Low/moderate	79 patients	N/A	- Systemic vascular resistance index - Cardiac index - Mean arterial pressure - Temperature - Central venous pressure	- Low SVR states post-CPB linked to vasoplegia
Janelle et al. (2000)^28^	Single-arm prospective	Graft perfusion response to CaCl_2_	High/low	20 patients	CaCl_2_ injection	- Flow velocity in internal mammary artery graft - Blood pressure, HR - Left atrial pressure	- CaCl_2_ transiently increases blood pressure and coronary perfusion pressure but significantly reduces internal mammary artery flow
Zaloga et al. (1990)^29^	Randomised control trial	Drug augmentation evaluation	High/high	12 patients	CaCl_2_ with epinephrine (adrenaline)	- Ionised calcium - Cardiac index - Mean arterial pressure	- CaCl_2_ raised mean arterial pressure, but not CI and blunted adrenergic responses of cardiac index from epinephrine
Royster et al. (1992)^30^	Randomised control trial	Calcium chloride and epinephrine post-CPB	High/high	40 patients	Calcium chloride bolus vs placebo ± epinephrine	- iCa, CO, MAP, PAP	- CaCl_2_ increases mean arterial blood pressure - CaCl_2_ did not decrease cardiac index immediately post-CPB
Johnston et al. (1992)^31^	Randomised control trial	CaCl_2_ vs ephedrine in CPB weaning support	High/moderate	36 patients	CaCl_2_, ephedrine, placebo	- RV volumes and ejection fraction via thermodilution catheters, MAP, HR, CI, iCa	- CaCl_2_ had insignificant effects on mean arterial pressure, right ventricular end-systolic volume, stroke volume, and ejection fraction during weaning from CPB
DeHert et al. (1997)^32^	Randomised control trial	Left ventricular function	High/moderate	20 patients	CaCl_2_ 5 mg/kg	- Left ventricular dP/dt, ejection fraction, MAP	- CaCl_2_ post-CPB transiently improved systolic function at the expense of an increase in ventricular stiffness
Belletti et al. (2026)^33^	Systematic analysis	Effect of intravenous CaCl_2_ administration in cardiac surgery	High/high	1278 patients	Intravenous calcium	- Mortality, adverse events - Hospital and ICU LOS - MAP, CO/CI, SVI, HR	- The most effective dosage of IV CaCl_2_ is 5–10 mg/kg - Peak effects observed within 5–10 min, and a subsequent return to baseline in most cases

CPB, cardiopulmonary bypass; CI, confidence interval; MAP, mean arterial pressure; SVR, systemic vascular resistance; ICU, intensive care unit; ECG, electrocardiogram; LOS, length of stay.

## 3 Results

### 3.1 Physiological rationale

Ionised calcium plays a critical role in myocardial contractility and vascular tone. In cardiac myocytes, it initiates contraction through depolarisation of the cardiac cell membrane, which opens L-type voltage-gated calcium channels and permits extracellular Ca^2+^ influx. This influx triggers calcium-induced calcium release (CICR) from the sarcoplasmic reticulum, which results in a tenfold rise in cytoplasmic Ca^2+^ concentration. Released Ca^2+^ ions then cause a conformational change in cardiac troponin C, thereby activating myocyte contraction.^34^ During normal cardiac contraction, peak cytosolic Ca^2+^ concentrations do not fully saturate the calcium-binding sites of contractile proteins, particularly troponin C. Consequently, variations in intracellular Ca^2+^ flux can thereby modulate the force of contraction.^35^ This property forms one of the fundamental physiological mechanisms through which myocardial contractility is regulated. Similarly, in vascular smooth muscle cells, ionised calcium binds to calmodulin, forming a complex that activates myosin light chain kinase (MLCK), which phosphorylates myosin and promotes actin-myosin interaction, resulting in vasoconstriction.^36^ A review in *Critical Care Clinics* described a direct relationship between iCa levels and arterial pressure in critically ill patients and reported that calcium administration was associated with short-term haemodynamic improvements such as increased arterial pressure and left ventricular stroke work index.^37^ Furthermore, postoperative ionised hypocalcaemia is frequently observed after CPB,^24^ although not at levels typically sufficient to cause cardiovascular compromise, pre- and intra-operative hypocalcaemia are significantly associated with higher vasoactive-inotropic scores and longer cross-clamp time.^38^ Mild hypocalcaemia within the first 24 h has also been shown to have a significant association with poorer short- and long-term prognosis and longer ICU and hospital stays.^38^^,^^39^

Post-CPB hypocalcaemia is believed to be due to several mechanisms: acute haemodilution, derangement of acid-base balance and plasma proteins, transfusion of citrate products, and inflammatory activation. Acute haemodilution from circuit priming reduces circulating albumin and electrolytes,^40^ while transfusion of citrate-containing blood products further lowers iCa through calcium–citrate complex formation. In addition, perioperative acid-base shifts significantly influence calcium binding to albumin, with alkalosis reducing biologically active iCa despite unchanged total calcium.^41^ The CPB-related inflammatory activation and transient alterations in parathyroid hormone regulation may further disrupt calcium homeostasis.^42^ Consequently, iCa frequently declines during and immediately after CPB, which may be associated with impaired myocardial contractility and vasoplegia in the early postoperative period. However, these changes often reflect transient dilutional, acid-base, and protein-binding shifts rather than true total body calcium deficiency.

Meta-analyses and small RCTs have suggested that prophylactic or early perioperative administration of levosimendan, a calcium sensitiser, in high-risk cardiac surgery patients may reduce low-cardiac-output syndrome, decrease vasopressor requirements, and shorten ICU length of stay.^43^^,^^44^ However, results remain inconsistent; while some studies have reported reductions in postoperative troponin release and improved cardiac output, larger multicentre RCTs have failed to show a mortality benefit.^45^^,^^46^ Consequently, guideline recommendations remain cautious, and practice varies widely across institutions.^47^ The relevance of levosimendan lies in its shared mechanistic pathway with calcium metabolism: both approaches optimise myocardial contractility, yet levosimendan enhances myocyte sensitivity to calcium rather than increasing calcium availability. Therefore, myocardial support may be achievable through the same pathway, either by modulating calcium sensitivity or by manipulating calcium concentrations directly. However, despite overlapping metabolic pathways, these interventions are mechanistically distinct, and findings from levosimendan studies cannot be extrapolated to calcium supplementation.

These mechanisms suggest that routine calcium supplementation could theoretically reduce the need for inotropic and vasopressor support without their potentially harmful side effects. Such a strategy may be clinically beneficial as lowering reliance on inotropes and vasopressors after cardiac surgery could limit associated drug-related complications such as arrhythmias, increased myocardial oxygen demand, and prolonged ICU stay.

### 3.2 In vitro and animal studies

In porcine ischaemia–reperfusion models, Kumar et al. (2009) showed impaired sarcoplasmic reticulum calcium uptake correlating with contractile dysfunction.^10^ Similarly, Rungatscher et al. (2013) showed that deep hypothermic circulatory arrest in Wistar rats induced myocardial calcium desensitisation, leading to impaired contractility and an increased requirement for inotropic support.^19^ Furthermore, Choi et al.^17^ demonstrated that calcium sensitisation improved systolic function and energy efficiency in postischaemic arrest in isolated rabbit hearts, although this may have the potential to exacerbate myocardial stunning. Collectively, these in vitro and animal studies suggest that postcardiac surgery results in both a state of irregular calcium metabolism and a period of relative hypocalcaemia. Therefore, calcium supplementation could theoretically provide a physiological intervention to overcome this desensitisation and hypocalcaemia to improve haemodynamic status. Grubb et al. demonstrated in six anaesthetised horses that calcium gluconate attenuated isoflurane-induced reductions in cardiac index, stroke index, and right ventricular contractility.^18^ While mechanistically informative, these findings remain experimental and may not directly translate to clinical outcomes in human cardiac surgical populations.

### 3.3 Weaning from cardiopulmonary bypass and ICU studies

Human research exploring calcium supplementation during CPB has produced mixed results. In a small study of 30 hypocalcaemic patients undergoing CPB, Auffant et al.^24^ reported that although post-CPB hypocalcaemia was common, routine calcium supplementation, both as boluses and infusions, did not significantly improve cardiac index, stroke volume, or vascular resistance beyond the first two postoperative hours. Conducted in 1981 without statistical power calculations, the study was likely underpowered and predates advances in contemporary cardiothoracic, anaesthetic, and intensive care practice. Similarly, a review by Lomivoratov et al.^48^ noted that while CPB was associated with reduced iCa, and CaCl_2_ at 5–15 mg/kg may transiently improve haemodynamic parameters, supplementation could potentially blunt catecholamine responsiveness and worsen haemodynamic instability. The ongoing ICARUS trial (818 patients) is evaluating whether administration of CaCl_2_ (15 mg/kg versus placebo) at the time of CPB weaning reduces postoperative inotrope requirements, however, it is unlikely to provide data beyond the early postoperative period in the ICU.^49^

Smaller RCTs have reported similarly transient haemodynamic effects. Royster et al.^30^ evaluated 40 patients during CPB weaning and found that CaCl_2_ with or without epinephrine (adrenaline), increased mean arterial pressure but did not improve cardiac index or augment catecholamine responsiveness. Although randomised, the 1992 study was small, lacked reported sample size calculations, and focused only on the immediate post-CPB period. An apparent improvement in cardiac index with calcium administration did not reach statistical significance, possibly reflecting limited power, and higher beta-blocker use in the treatment arm may have confounded results. Likewise, Shapira et al.^27^ reported early improvements in mean arterial pressure, stroke volume index, and SVR after CaCl_2_ injection in a small sample of 26 patients, but these effects dissipated within minutes and were assessed only in the first 10 min after CPB weaning, and therefore not extrapolatable to an ICU setting. Similarly, De Hert et al.^37^ observed transient improvement in systolic function offset by increased ventricular stiffness in just 20 patients, with effects resolving within 10 min and no data beyond 20 min post-CPB. Collectively, these studies are small, dated, and confined to the immediate peri-CPB period, limiting extrapolation to sustained ICU outcomes.

Other studies suggest neutral or potentially adverse effects. Zaloga et al.^29^ examined 12 patients 24 h post-CPB in the ICU and found that coadministration of calcium and epinephrine may blunt epinephrine's inotropic effects, producing only a modest increase in blood pressure without improvement in cardiac index. Similarly, Johnston et al.^31^ reported no meaningful improvement in right ventricular performance with CaCl_2_, even in hypocalcaemic patients, and found it inferior to ephedrine for augmenting arterial blood pressure augmentation during CPB weaning. Although blood pressure appeared to rise more with calcium than placebo, this was not statistically significant in the small (n = 36) cohort and no power calculation was reported. Lastly, Myles et al.^26^ demonstrated that postoperative vasoplegia syndrome (“low SVR syndrome”) post-CPB was not more frequent in patients taking preoperative calcium antagonists, suggesting that calcium supplementation would be of little benefit in the development of postoperative vasoplegic syndrome. This finding, however, pertained to vasoplegia specifically and does not exclude possible benefit in other contributors to postoperative haemodynamic instability (e.g. myocardial stunning, relative hypovolaemia).

Beyond the perioperative cardiac setting, critical care data on calcium supplementation are similarly mixed. Newman et al.^50^ in a meta-analysis of 41 case reports and 3 case series, identified reversible cardiac dysfunction in the context of significant ionised hypocalcaemia, although no preventive threshold could be defined, left ventricular ejection fraction correlated with both total and ionised calcium. In contrast, Egi et al.^25^ examined iCa in 7024 ICU patients and found that only extreme values (<0.8 mmol/L or >1.4 mmol/L) were independently associated with ICU and hospital mortality, whereas iCa within a broad physiological range was not independently associated with ICU or hospital mortality.

Studies investigating paediatric populations in this area have demonstrated similarly inconsistent haemodynamic responses to intravenous calcium supplementation. Murdoch et al. evaluated the efficacy of a calcium chloride (CaCl_2_) bolus in 12 paediatric cardiothoracic patients 6 h following cardiac surgery. Despite the small sample size, the authors reported statistically significant increases in arterial pressure, which were attributed to an increased SVRI and a corresponding decrease in CI.^20^ These findings contrast with those observed in the nonsurgical paediatric cardiac intensive care population. In a retrospective analysis of 68 patients, Averin et al. showed that CaCl_2_ infusions resulted in a sustained improvement in cardiac output for up to 6 h, with statistically significant improvements in lactate and urine output, implying better organ perfusion.^21^ This outcome was corroborated by Karki et al. who found that CaCl_2_ augmented markers of cardiac output and organ perfusion when administered with vasopressin in children presenting with acute heart failure.^22^ Although the literature suggests that CaCl_2_ may be beneficial from a haemodynamic perspective, this improvement may not translate to patient-centred outcomes. Kimura et al. found higher iCa levels to be independently associated with increased paediatric ICU length of stay, but only at levels >1.30 mmol/L.^23^ Only one of these studies published data on the primary pathology of their participants, and given that most paediatric cardiac surgery and cardiac ICU involves congenital heart disease, the findings may not be directly generalisable. Moreover, paediatric populations are inherently heterogeneous, encompassing neonates, infants, and older children, with variations in CBP strategies and differing perioperative transfusion exposure that may contribute to differences in calcium kinetics and the overall risk–benefit profile of calcium supplementation.

The most comprehensive evidence to date comes from the 2026 systematic review and meta-analysis by Belletti et al.^33^ which evaluated the safety and efficacy of intravenous calcium in a cardiac surgical population, that included 22 studies (n = 1278 patients), of whom 809 received calcium. Calcium administration was associated with a modest but statistically significant reduction in heart rate and a transient increase in mean arterial pressure (mean difference of 6.45 mmHg), without significant change in SVR or cardiac index/output. Importantly, the review highlighted the lack of robust RCTs examining clinically relevant outcomes such as mortality, adverse events, vasopressor duration, or ICU length of stay.

### 3.4 Theoretical risks of calcium supplementation

Theoretical risks of routine calcium supplementation, though rare, warrant consideration. To our knowledge, the only large RCT assessing clinical outcomes and calcium administration is the COCA trial^51^, which examined the co-administration of CaCl_2_ with adrenaline in out-of-hospital cardiac arrest patients. Although conducted in a different population from cardiac surgical patients, this trial failed to demonstrate benefit and was terminated early due to safety concerns and lack of efficacy. Notably, the signal towards harm persisted at 1-year follow-up,^52^ highlighting the need for caution when extrapolating calcium use beyond clearly defined indications.

Excess calcium can exacerbate intracellular accumulation, potentially aggravating ischaemic injury or reducing flow in grafted vessels.^16^ Indeed, calcium channel antagonists have long been used to relieve coronary arteriolar vasospasm.^28^ In animal models, elevated intracellular calcium has been associated with moderate cytosolic calcium overload, and heightened calcium sensitivity can worsen myocardial stunning.^19^ Rarely, calcium administration has been implicated in the “stone heart” phenomenon in digoxin toxicity, though its role in this syndrome remains debated.^53^ With zero reported cases in the most recent systematic review of calcium supplementation in cardiac surgery,^33^ this risk appears to be mainly theoretical. Other recognised risks include hypercalcaemia overshoot and tissue necrosis from extravasation.^54^ Nonetheless, in contemporary practice, these risks appear minimal. Digoxin is commonly withheld or reassessed in the immediate postoperative period following cardiac surgery due to apparent increased sensitivity to digoxin toxicity and an increased risk of arrhythmias^55^, and ionised calcium levels are closely monitored with regular arterial blood gases in the postoperative period to mitigate risks of excess accumulation.

### 3.5 Current knowledge gaps

This narrative review highlights the ongoing uncertainty surrounding the role of calcium administration in patients undergoing major cardiothoracic surgery. While calcium is widely recognised as a key mediator of myocardial contractility and vascular tone, the evidence supporting its use as a therapeutic adjunct to reduce vasopressor or inotrope requirements remains limited and inconsistent.

Most existing studies have focused on calcium administration during CPB or in the immediate CPB period. They consistently suggest that calcium supplementation during CPB weaning or in the immediate post-CPB period can transiently improve haemodynamic parameters such as mean arterial pressure, SVR, and left ventricular stroke work index. However, these effects are often short-lived, the published studies are small, and there is little evidence addressing outcomes in the later postoperative/ICU phase or patient-centred outcomes such as mortality, ICU or hospital length of stay, or complications.

### 3.6 Suggestions for future studies

Existing data contrast with the strong physiological rationale for calcium supplementation. Given its essential role in excitation-contraction coupling and vascular smooth muscle contraction, increasing extracellular calcium availability should theoretically enhance contractility and vascular tone. The analogy with levosimendan is notable: while levosimendan enhances responses to endogenous calcium, supplementation increases calcium availability directly. This pattern reinforces the notion that modifying calcium homeostasis by supplementation may improve short-term haemodynamics and could reduce vasopressor or inotropic use and potentially shorten the ICU stay. Additionally, the absence of specific guideline recommendations on calcium supplementation contrasts with detailed consensus statements regarding vasopressor therapy after cardiac surgery.^11^ This lack of standardisation likely explains the wide variation in practice observed internationally, with calcium use often dictated by local tradition rather than robust evidence. As the largest contemporary RCT in this area and having recently completed recruitment, the ICARUS trial aims to clarify the role of calcium supplementation in facilitating separation from CPB, primarily by assessing vasopressor–inotrope requirements within the first 30 min. Although the trial will also evaluate vasopressor–inotrope use on postoperative day 1 and 30-day mortality as secondary outcomes, it does not address longer-term or ICU-focused outcomes. Specifically, it does not examine the impact of additional calcium administration in the ICU, vasopressor–inotrope requirements beyond the first postoperative day, or other clinically relevant endpoints such as length of ICU stay.^49^

The current evidence base is heterogeneous, with studies reporting benefit, no effect, and in some cases possible harm, such as in the COCA trial and subsequent follow-up.^51^^,^^52^ Though RCTs remain the gold standard for causal inference, in this context, a target trial emulation (TTE) may offer an informative complementary approach, especially since calcium administration has wide and varied use in routine postoperative practice. This provides an opportunity to examine different treatment strategies and explore potential heterogeneity in treatment effects using real-world data. Additionally, if the true effect on outcomes such as survival is modest, randomised trials designed to detect mortality differences would likely require prohibitively large sample sizes, be logistically complex, and may expose patients to harm. If the TTE provides an estimate of the effect of calcium supplementation in patients undergoing cardiac surgery, these findings could inform sample size calculations and feasibility assessments for future RCTs.

Future research should focus on large, contemporary, multicentre RCTs or TTEs conducted within modern perioperative and ICU care. Such studies should evaluate not only short-term haemodynamic endpoints but also more ICU-relevant and patient-centred outcomes including vasopressor duration, ICU length of stay, and mortality, to define the true role of calcium supplementation in modern cardiac surgery practice.

### 3.7 Limitations

As a narrative review, this article is subject to selection and publication bias, and the studies discussed are heterogeneous in design, patient populations, and outcomes. Most available evidence is from older, small, or peri-CPB studies, with no evidence existing examining the total duration of vasopressor use, limiting the ability to generalise findings to modern ICU settings. The absence of a systematic methodology means some relevant studies may not have been included. It is important to acknowledge that multiple formulations of vasopressors and inotropes are commercially available, differing in concentration, excipients, and delivery mechanisms. These variations can influence pharmacodynamic responses and may limit the direct comparability of results across clinical studies and institutional protocols.^56^^,^^57^ The use of vasopressors is discussed within the study in relation to haemodynamic effects and calcium responsiveness, with norepinephrine (noradrenaline) and/or vasopressin being the recommended agents.^11^

## 4 Conclusion

Calcium is commonly administered during CPB weaning and to support blood pressure and cardiac output after cardiothoracic surgery. While transient haemodynamic improvements have been reported, there is no robust evidence that supplementation reduces postoperative inotrope or vasopressor requirements beyond the peri-CPB phase. Most existing studies are small, uncontrolled, or proof-of-concept in design, and no RCTs have examined ICU-relevant or patient-centred outcomes. Importantly, much of the foundational evidence derives from an era preceding modern advances in myocardial protection, CPB techniques, anaesthetic management, and ICU care. Furthermore, contemporary cardiac surgical populations tend to be older and comorbid, further limiting the generalisability of historical data and highlighting the need for updated research evaluating calcium therapy within modern practice paradigms.

High-quality, multicentre trials primarily focusing on calcium supplementation and total inotrope-vasopressor duration, with secondary outcomes examining mortality, ICU, and hospital length-of-stay and organ dysfunction across the entire postoperative ICU period are needed to establish its role in contemporary perioperative and critical care.

## CRediT authorship contribution statement

Adam Miller: Conceptualisation, Methodology, Writing – Original Draft Preparation, Jessica Omassoli: Conceptualisation, Methodology, Writing - Original Draft Preparation, Joanna Simpson: Supervision, Writing – Review and Editing, Kyle Brooks: Writing – Review and Editing, Aubrey Almeida: Writing – Review and Editing, Ryan Ruiyang Ling: Writing – Review and Editing, Ashwin Subramaniam: Supervision, Conceptualisation, Methodology, Writing – Original Draft Preparation, Review and Editing.

## Funding

This research did not receive any specific grant from funding agencies in the public, commercial, or not-for-profit sectors.

## Conflict of interest

The authors declare the following financial interests/personal relationships which may be considered as potential conflict of interests: One of the coauthors, Ashwin Subramaniam, is part of the Editorial Board and an associate editor for Critical Care and Resuscitation but will not be involved in any decisions regarding the publication of this manuscript. If there are other authors, they declare that they have no known competing financial interests or personal relationships that could have appeared to influence the work reported in this paper.
